# Vaccine Effects on In-hospital COVID-19 Outcomes

**DOI:** 10.1097/EDE.0000000000001877

**Published:** 2025-06-17

**Authors:** Bronner P. Gonçalves, Piero L. Olliaro, Peter Horby, Benjamin J. Cowling

**Affiliations:** From the a ISARIC, Pandemic Sciences Institute, University of Oxford, Oxford, United Kingdom; b Faculty of Health and Medical Sciences, University of Surrey, Guildford, United Kingdom; c WHO Collaborating Centre for Infectious Disease Epidemiology and Control, School of Public Health, Li Ka Shing Faculty of Medicine, The University of Hong Kong, Hong Kong Special Administrative Region, People’s Republic of China.

**Keywords:** Causal inference, Disease progression, Post-treatment variable, Potential outcomes, Principal stratification, Vaccination

## Abstract

Here, we posit that studies comparing outcomes of patients hospitalized with COVID-19 by vaccination status are important descriptive epidemiologic studies, but they contrast two groups that are not comparable with regard to causal analyses. We use the principal stratification framework to show that these studies can estimate a causal vaccine effect only for the subgroup of individuals who would be hospitalized with or without vaccination. Further, we describe the methodology for, and present sensitivity analyses of, this effect. Using this approach can change the interpretation of studies only reporting the standard analyses that condition on observed hospital admission status—that is, analyses comparing outcomes for all hospitalized COVID-19 patients by vaccination status.

During the first years of the coronavirus disease 2019 (COVID-19) pandemic, various studies investigated associations between vaccination against the severe acute respiratory syndrome coronavirus 2 (SARS-CoV-2) and disease outcomes (e.g., death) of hospitalized COVID-19 patients.^1–5^ For example, two studies^1,2^ in the United States found that among patients admitted with SARS-CoV-2 infection, the risk of death was lower in those with a history of vaccination compared to those with no previous SARS-CoV-2 vaccination. A Norwegian study, on the other hand, reported higher in-hospital mortality in vaccinated individuals compared to unvaccinated individuals in unadjusted analyses, and no difference in adjusted analyses.^3^

These hospital-based studies that only recruit COVID-19 patients (henceforth, in-hospital outcome vaccine studies) are important as they allow the characterization of disease presentation and outcome in hospitalized patients by vaccination status, which is useful for clinicians and healthcare planning. Here, however, we argue that these analyses do not have a valid causal interpretation. In fact, these studies condition, by design, on a post-treatment variable (here, vaccination is the treatment variable, and hospitalization is the post-treatment variable), which introduces a form of selection bias. In other words, the comparison of in-hospital outcomes by vaccination status does not correspond to a contrast of potential outcomes for the same set of individuals,^6^ which is a requirement for average causal effects. It is worth highlighting that here we do not discuss hospital-based studies that also recruit hospitalized control patients, that is, hospitalized patients who are not infected with SARS-CoV-2 (e.g., test-negative or case–control designs), and that estimate vaccine efficacy against SARS-CoV-2-associated hospitalization.

The paper is organized as follows. In the next section, we describe the analysis of these studies using the potential outcomes framework^7^ and the principal stratification framework.^8^ Then, we present sensitivity analyses for a type of effect that can be defined in such studies (principal effect, described in the next section). We conclude with a discussion and comment on similar analyses for booster vaccine doses.

## NOTATION AND DEFINITION OF PRINCIPAL EFFECT

Let V denote SARS-CoV-2 vaccination (1 = vaccinated, 0 = not vaccinated), and H, the post-treatment variable corresponding to COVID-19-related hospitalization (1 = hospitalized with COVID-19, 0 = not hospitalized with COVID-19). Further, assume that the outcome, Y, is in-hospital death (1 = death, 0 = survival). Then, the quantities that are often reported in such studies are Pr(Y=1|H=1,V=1) and Pr(Y=1|H=1,V=0). To explain why a comparison of these quantities does not correspond to a causal estimand, we also define the potential outcomes variables $H^{v}$ and $Y^{v}$, which represent potential hospitalization and potential in-hospital outcome under the exposure value v. Given these variables, under the principal stratification framework,^8^ the population can be partitioned into four strata based on the joint potential hospitalization values under two vaccination levels: $\left\{\;j:H_{j}^{1}=1,H_{j}^{0}=1\right\}$, $\left\{\;j:H_{j}^{1}=1,H_{j}^{0}=0\right\}$, $\left\{\;j:H_{j}^{1}=0,H_{j}^{0}=1\right\}$, $\left\{\;j:H_{j}^{1}=0,H_{j}^{0}=0\right\}$; here j represents different individuals. Below, we refer to these strata, respectively, as “doomed,” “harmed,” “protected,” and “immune.”

Now, in in-hospital outcome vaccine studies, individuals who are hospitalized and vaccinated are, by the definition of these strata, either in the “doomed” or “harmed” stratum; similarly, hospitalized unvaccinated individuals can only be members of two principal strata, “doomed” or “protected” stratum. Thus, the observed groups defined by (H=1,V=1) and (H=1,V=0) only overlap in the “doomed” stratum. For this reason, the comparison of in-hospital outcomes by vaccination status conditional on hospitalization would only have a causal interpretation if analysis were restricted to individuals who would be hospitalized with COVID-19 regardless of vaccination history. In this case, the estimand that compares frequencies of in-hospital outcomes for this principal stratum (“doomed” stratum) quantifies a principal effect.

Using notation similar to,^9^ this effect, expressed as relative risk difference ($VE_{in-h}$, which represents in-hospital vaccine efficacy in the “doomed” stratum), can be formally defined as:


$$\begin{array}{l} VE_{in-h}=1-\frac{\phi_{1.}}{\phi_{.1}}, \end{array}$$

where $\phi_{1.}=\mathrm{Pr}(Y^{1}=1|H^{1}=1,H^{0}=1)$, and $\phi_{.1}=\mathrm{Pr}(Y^{0}=1|H^{1}=1,H^{0}=1)$.

An important difficulty in using this approach, however, is that, in general, it is not possible to identify to which principal stratum an individual belongs, as we only observe one of the potential outcomes (either $H^{1}$ or $H^{0}$), making this effect nonidentifiable without additional assumptions.

## SENSITIVITY ANALYSES FOR VACCINE EFFECTS ON IN-HOSPITAL COVID-19 OUTCOMES

In this section, we use methods described by Hudgens and Halloran^9^ for sensitivity analyses of principal effects to investigate plausible vaccine effects on in-hospital COVID-19 outcomes in the “doomed” stratum.

If we assume that vaccination does not cause COVID-19-related hospitalization for any individual in the population, so that no individual has the joint potential hospitalization outcome ($H^{1}=1,H^{0}=0$), all vaccinated hospitalized individuals are members of the “doomed” stratum and thus directly inform the distribution of the in-hospital outcome under vaccination in this stratum. However, among unvaccinated hospitalized patients, there are both individuals in the “doomed” stratum and “protected” stratum ($H^{1}=0,H^{0}=1$). The frequency of the outcome Y in unvaccinated hospitalized patients is thus a function of the in-hospital outcome under no vaccination for these two strata (“doomed” and “protected”; see equation 15 in^9^). Although it is not possible to determine to which of these principal strata unvaccinated patients belong, in studies that collect information on efficacy against hospitalization and on in-hospital outcomes for the same population, additional assumptions allow sensitivity analyses for principal effects.

To investigate possible magnitudes of principal effects in these studies, we use formulas that encode assumptions on selection, which here corresponds to hospitalization (see Section 4 in^9^). Although these methods were developed for postinfection outcomes, we apply the approach to in-hospital outcomes. Briefly, the odds ratio (β) of having a more severe in-hospital outcome under no vaccination in the “doomed” versus the “protected” stratum is assumed known and varied. Formally, β is:


$$\begin{array}{l} \beta =\frac{\frac{\mathrm{Pr}(Y^{0}=1|H^{1}=1,H^{0}=1)}{\left(1-\mathrm{Pr}(Y^{0}=1|H^{1}=1,H^{0}=1)\right)}}{\frac{\mathrm{Pr}(Y^{0}=1|H^{1}=0,H^{0}=1)}{\left(1-\mathrm{Pr}(Y^{0}=1|H^{1}=0,H^{0}=1)\right)}} \end{array}$$

Note that β=1 is consistent with no selection bias.

The Figure presents the results of calculations that assume 90% efficacy against hospitalization, and two different values for β. For a scenario similar to that in the study by Mielke and colleagues,^1^ where 10.3% of vaccinated and 12.8% of unvaccinated hospitalized patients died, these assumptions are consistent with $VE_{in-h}$ of 19.5% when β=1, and with $VE_{in-h}$ of 71.6% when β=5. More generally, depending on the real, but in practice unknowable, value of β, studies that observe similar or higher frequencies of severe in-hospital outcomes in vaccinated versus unvaccinated individuals are not incompatible with a protective effect on in-hospital disease progression in the “doomed” stratum; that is, for these individuals, vaccination could be protective against in-hospital outcome even if Pr(Y=1|H=1,V=1)>Pr(Y=1|H=1,V=0).

**Figure.**
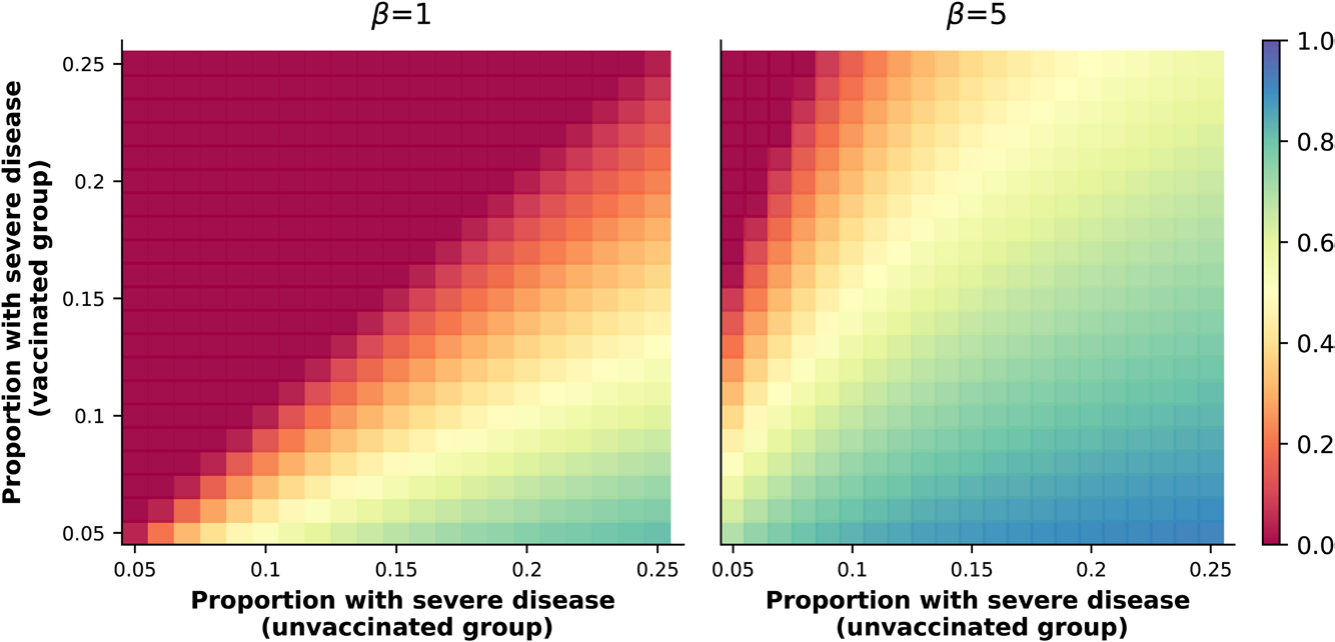
Sensitivity analyses. The figure shows principal effects (the different colors indicate the magnitude of $VE_{in-h}$ in the “doomed” stratum; scale is shown on the right) in different scenarios that assume 90% vaccine efficacy against COVID-19 hospitalization and the odds ratio, β, values of 1 (left panel) and 5 (right panel). X- and y-axes correspond to proportions of unvaccinated and vaccinated patients with the more severe in-hospital outcome, Pr(Y=1|H=1,V=0) and Pr(Y=1|H=1,V=1). Calculations used equations 8, 15, and 28 in the manuscript by Hudgens and Halloran (see main text). Notice that in this figure, rather than using data from a single study, we perform calculations over a range of plausible values for the frequencies of the in-hospital outcome. The color scale only ranges from 0 to 1, and negative principal effects in the “doomed” stratum are represented with the same color as no effect (dark red). In the *eAppendix*; https://links.lww.com/EDE/C244 we present sensitivity analyses for different values of β (**eFigure**; https://links.lww.com/EDE/C244), and present additional information on the calculations performed to generate these figures.

In general, the assumption that, under no vaccination, the odds of severe in-hospital outcome are higher in the “doomed” stratum compared to the “protected” stratum seems plausible if these strata represent distinct comorbidity profiles that, for example, determine whether the protection afforded by vaccination is sufficient to prevent hospitalization and that are also associated with in-hospital prognosis. Finally, note that another assumption here is the independence of treatment (vaccination) assignment and potential post-treatment and outcome values. While this is a reasonable assumption in randomized trials, in many observational studies it might not be justified; for instance, in early phases of the pandemic, with limited vaccine availability, individuals at risk of severe outcomes (those with comorbidities or who were older) were preferentially vaccinated.

## DISCUSSION

The discussion above has implications for both analyses and interpretation of in-hospital outcome vaccine studies. First, it will facilitate appropriate interpretation of these studies by epidemiologists and policy makers, and hence prevent unwarranted decision-making (e.g., decisions that disregard the fact that these analyses do not correspond to standard vaccine effectiveness analyses). Second, when the scientific question relates to understanding protective mechanisms and estimating vaccine effects on disease progression, these studies could be useful, but for this type of inference, additional assumptions and sensitivity analyses are needed, and the approach illustrated here will be useful to investigators.

Note, however, that although knowing whether vaccination has an effect on disease severity even when it does not prevent disease occurrence improves our mechanistic understanding, policy decisions often require information on total vaccine effects (i.e., that do not condition on hospitalization). Furthermore, causal insights from principal effects would only apply to a nondiscernible fraction of the population. Still, as mentioned above, in-hospital outcome vaccine studies are also important descriptive studies that can inform allocation of resources in future SARS-CoV-2 waves or pandemics.

Note that even though we focused, for simplicity, on comparing vaccination versus no vaccination, a relevant factor when considering principal effects of vaccines is the dose-dependent nature of induced immunity and its possible impact on the relative frequencies of principal strata. For instance, in studies that compare outcomes between fully vaccinated individuals who received a booster dose and vaccinated individuals who did not receive a booster dose, it is plausible that the fractions of the populations in the “doomed” stratum are smaller than those in comparisons between fully vaccinated individuals without booster dose versus unvaccinated individuals. The implication is that, if in-hospital outcome vaccine studies of patients with multiple vaccine doses are to be interpreted causally, for example, after sensitivity analyses under different selection models, the principal effect in the nondiscernible “doomed” stratum would be relevant for a small fraction of the population.

## Supplementary Material
