# Supplementary material for: Vaccine Effects on In-hospital COVID-19 Outcomes
Source: Epidemiology. 2025 Jun 17;36(5):646–9. doi: 10.1097/EDE.0000000000001877 (PMC12303245; doi:10.1097/EDE.0000000000001877)
Supplement: Supplementary file 1 [file ede-36-646-s001.pdf]

eAppendix

Table of Contents

Sensitivity analysis (Supplemental Methods)
eFigure

Sensitivity analysis (Supplemental Methods)

This section uses the notation presented in the main text and provides additional information on the sensitivity analysis. For that, we introduce parameters similar to those in the study by Hudgens and Halloran [1]:

$$\phi_{.1} = \Pr(Y^0 = 1 | H^1 = 1,\ H^0 = 1)$$
$$\gamma \quad = \Pr(Y^0 = 1 | H^1 = 0,\ H^0 = 1)$$

where $\phi_{.1}$ is the probability of occurrence of the in-hospital outcome under no vaccination in the "doomed" stratum, and γ is the same probability for the "protected" stratum. We can thus express β, introduced in the main text, using these parameters:

$$\beta = \frac{\phi_{.1}/(1-\phi_{.1})}{\gamma/(1-\gamma)}$$

(Equation 1)

Moreover, the following relation is used (see Section 3 in [1]):

$$\Pr(Y^0 = 1 | H^0 = 1) = \gamma\, VE_{hosp} + \phi_{.1}(1 - VE_{hosp})$$

(Equation 2)

Here, VE_{hosp} denotes the vaccine efficacy for hospital admission (rather than for the in-hospital outcome). In words, the probability of the in-hospital outcome under absence of vaccination conditional on the potential value H^0 being 1 depends on the probability of the in-hospital outcome for the "doomed" and "protected" strata, with VE_{hosp} and $(1 - VE_{hosp})$ corresponding to relative weights.

Thus, to calculate VE_{in-h}, we solve *Equation 1* for γ or $\phi_{.1}$ and substitute into *Equation 2.* Assuming that VE_{hosp} and β are known, we solve a quadratic equation and can then calculate

$$VE_{in-h} = 1 - \frac{\phi_{1.}}{\phi_{.1}}$$

Note that under consistency, exchangeability and monotonicity ($H^1 \leq H^0$ for all individuals), both $\Pr(Y^0 = 1|H^0 = 1)$ and $\phi_{1.}$ are observable in the data.

It is worth noting that when β is assumed to be below 1 (e.g. 0.5), VE_{in-h} will more often be negative. This is explained by the fact that when $\beta < 1$, the probability of in-hospital outcome under no vaccination would be higher for the "protected" versus the "doomed" stratum ($\gamma > \phi_{.1}$) and that the parameter $\phi_{.1}$ is used in the definition of VE_{in-h}.

Finally, as the approach described in [1] was for post-infection outcomes, rather than for in-hospital outcomes, the interpretation of parameters needs to be adapted to our context. For instance, while here we use VE_{hosp}, vaccine efficacy against hospitalisation, in [1], the authors use VE_S for vaccine efficacy against infection. Furthermore, VE_{in-h} refers to individuals who would be hospitalised regardless of exposure status, and not to those who would be infected regardless of exposure status.

eFigure. Sensitivity analyses for additional scenarios defined based on the parameter β. The structure of this figure is similar to that of the **Figure** (main text). The left panel uses $\beta = 2.5$, and the right panel uses $\beta = 10$. In this figure, we assume the same vaccine efficacy against hospitalisation (90%) as in the **Figure**.

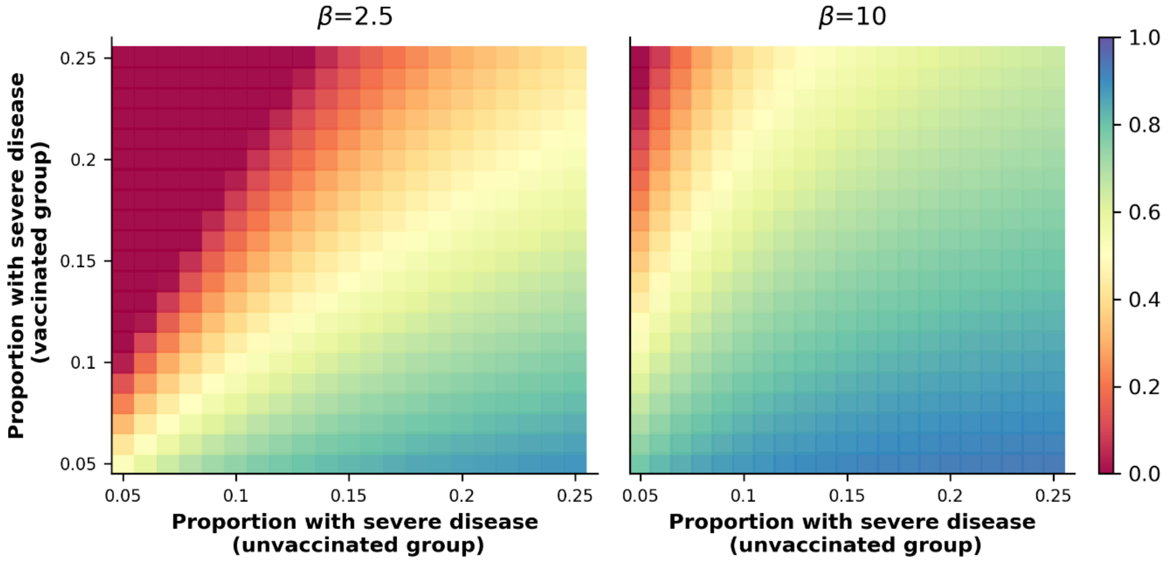

References

1. Hudgens, M.G. and M.E. Halloran, *Causal Vaccine Effects on Binary Postinfection Outcomes.* J Am Stat Assoc, 2006. **101**(473): p. 51-64.
